# New-Onset Movement Disorders in COVID-19: Much Ado about Nothing?

**DOI:** 10.5334/tohm.644

**Published:** 2021-07-28

**Authors:** Melissa J. Nirenberg

**Affiliations:** 1 Icahn School of Medicine at Mount Sinai, NY, USA

**Keywords:** COVID-19, movement disorder, hyperkinetic, myoclonus, tremor, parkinsonism, ataxia

## Abstract

This is an editorial commenting on the paper by Brandão and colleagues [Brandão PRP, Grippe TC, Pereira DA, Munhoz RP, Cardoso F. New-Onset Movement Disorders Associated with COVID-19. Tremor and Other Hyperkinetic Movements. 2021; 11(1): 26. DOI: *http://doi.org/10.5334/tohm.595*]

## Introduction

Coronavirus disease-19 (COVID-19), caused by SARS-CoV-2, has been associated with a variety of neuropsychiatric manifestations. Most have been attributed to indirect mechanisms: these include toxic-metabolic complications leading to encephalopathy, hypercoagulability leading to stroke, or immune-mediated mechanisms leading to acute disseminated encephalomyelitis (ADEM), transverse myelitis, or Guillain-Barré syndrome [1]. Despite concerns about potential direct central nervous system (CNS) effects of COVID-19, only a few studies to date have supported this possibility. Neuroimaging studies have been remarkable mainly for ischemic disease, hemorrhagic complications, or findings of inflammatory or immune-mediated processes (e.g., ADEM). Spinal fluid analysis has only rarely shown evidence of SARS-CoV-2 in the CNS [1,2]. In cases of COVID-19 that have come to autopsy, acute and subacute neuropathological changes have largely paralleled the observed spectrum of clinical findings, with features consistent with ischemic stroke, brain hemorrhage, hypoxia, other complications of critical illness, and demyelinating diseases such as ADEM [3,4,5]. Thus, despite the high prevalence of neuropsychiatric complications of COVID-19, current clinical, imaging, biomarker, and neuropathological data suggest that direct CNS involvement of SARS-CoV-2 is uncommon.

In the movement disorders literature, a specific concern has been raised about whether COVID-19 might cause a hypokinetic disorder similar to the epidemic of postencephalitic parkinsonism that developed a century earlier in the setting of the Spanish flu pandemic (though the influenza virus was not proven to be causative) [6,7]. The initial presentation of encephalitis lethargica was with fever, oculomotor disturbances, and movement disorders; this was followed by delayed-onset postencephalitic parkinsonism [8]. A number of factors have contributed to speculation about a potential relationship between SARS-CoV-2 and parkinsonism, including both the frequency and prominence of olfactory dysfunction in COVID-19 and Parkinson’s disease (PD); and the binding of the SARS-CoV-2 to angiotensin-converting enzyme 2 (ACE-2) receptors in the nasal mucosa (a potential site of entry and retrograde transsynaptic spread of the virus, similar to the proposed mechanism of spread in encephalitis lethargica) [6]. Other coronaviruses are known to be neuroinvasive, potentially entering the CNS and spreading transsynaptically through this pathway, making this a biologically plausible hypothesis.

Concerns about potential direct involvement of COVID-19 in the CNS causing parkinsonism or other movement disorders is of critical clinical and scientific importance. Most of the movement disorders literature, however, has focused on outcomes of COVID-19 infection in patients with preexisting movement disorders such as PD. In the manuscript by Brandão and colleagues [9], the authors address this important knowledge gap by performing a comprehensive review of all published cases of new-onset movement disorders in COVID-19 from the beginning of the pandemic through January 2021.

## Results

In total, the authors identified only 93 cases of new-onset movement disorders in COVID-19, derived from 44 published manuscripts spanning more than a year of this global pandemic. Even this small number of cases was derived using a broad definition of movement disorders that included isolated oculomotor disorders.

Of the subjects with COVID-19-related movement disorders who were described in this review, hyperkinetic movements predominated. Almost two-thirds of cases were of myoclonus, a nonspecific movement disorder that frequently occurs in hospitalized patients related to medication use and a variety of toxic-metabolic conditions. Action/postural tremor, another movement disorder that can be triggered or exacerbated by medications and toxic-metabolic conditions, was also present in about 11% of cases. Both myoclonus and tremor were closely associated with encephalopathy as well as the use of potentially causative medications (e.g., serotonergic medications or opioids).

Ataxia was the second most common new-onset movement disorder, after myoclonus, and was present in almost 40% of cases. The spectrum of causes of ataxia was multifactorial, including immune-mediated disorders (cerebellitis and Miller-Fisher syndrome). Many subjects had more than one type of movement disorder, and oculomotor abnormalities were often seen in combination with ataxia or myoclonus.

In contrast with the high frequency of hyperkinetic movements, there were only 5 of cases of a new-onset hypokinetic movement disorder (akinetic-rigid syndrome). These included 3 cases of new-onset parkinsonism and 2 cases of catatonia.

## Discussion

Perhaps the most interesting finding of this study is just how few descriptions there have been of new-onset movement disorders in COVID-19 [10]. Despite the high worldwide prevalence of the disease and intense interest in characterizing its protean clinical manifestations, the authors identified fewer than 100 published cases.

While concerns have focused on potential COVID-19-related parkinsonism, the findings of this review suggest that hyperkinetic movement disorders predominate, and hypokinetic disorders are rare. Myoclonus was most the most commonly observed movement disorder, indicating that movement disorders – like other neurological manifestations of COVID-19 – may be primarily related to toxic-metabolic complications (e.g., hypoxia, sepsis, organ failure, medications) rather than direct manifestations of the disease. Other observed new-onset hyperkinetic disorders were also attributable to indirect mechanisms, including three observed cases of functional movement disorders.

Because the data presented in this review was largely derived from individual case reports and case series, it is not possible to determine whether the frequency of myoclonus, tremor, or other observed movement disorders is different in COVID-19 versus other viral illnesses of similar disease severity. Myoclonus, for example, may have been significantly underreported because it was not felt to be sufficiently novel to warrant publication; it also may have been eclipsed by other disease manifestations.

In contrast with the high proportion of hyperkinetic movements, there were very few reported new-onset hypokinetic disorders. The authors identified only 3 published cases of new-onset parkinsonism (all with evidence of presynaptic dopamine depletion on functional neuroimaging) and 2 of catatonia. It is common for preexisting parkinsonism to first be recognized in the setting of intercurrent illness [11], and this might potentially explain some (or all) of these cases of “new-onset” parkinsonism. The other 2 cases of new-onset akinetic-rigid syndromes were of catatonia (which notably can be part of the presentation of encephalitis lethargica). Overall, however, the findings are highly reassuring, particularly given multiple lines of converging evidence to suggest that direct CNS involvement of SARS-CoV-2 is rare.

In conclusion, this comprehensive review of the literature suggests that movement disorders in COVID-19 are mainly epiphenomena, related to toxic-metabolic dysfunction, vascular disease, inflammatory or immune-mediated disorders, or other nonspecific complications of medical illness. While the published literature shows no evidence that COVID-19 causes postencephalitic parkinsonism, longitudinal follow-up is warranted to evaluate for potential long-term movement disorders complications. Delayed-onset movement disorders after COVID-19-related stroke, for example, may not manifest until years in the future.
